# Supplementary material for: Nurses’ knowledge, perceived challenges, and recommended solutions regarding premature infant care: A mixed method study in the referral and tertiary hospitals in Dar es salaam, Tanzania
Source: PLoS One. 2023 Mar 29;18(3):e0281200. doi: 10.1371/journal.pone.0281200 (PMC10057798; doi:10.1371/journal.pone.0281200)
Supplement: S1 File — (DOCX) [file pone.0281200.s001.docx]

**Supportive Information 1(S1): Self-administered questionnaire English version**

Questionnaire #.................Name of your facility…………………………………………

**Circle and fill in appropriately**

**Gender:**

Male

Female

**Age:**

**Cadre of health worker:**

**Highest level of education you hold?**

Certificate

Diploma

degree

**How many years have you served as a neonatal nurse in caring for the premature LBW babies?** (Completed years)

**When did you last attend a training or workshop on premature LBW newborn care?**

**Essential Newborn Care**

**1. What is the definition of low birth weight for newborn child?**

**a)** Weight is less than 3000 grams,

**b) Weight is less than 2500 grams,**

**c)** Weight is less than 1500 grams,

**d)** Weight is less than 1000 grams,

**2. Shortly after birth a premature LBW newborn baby should be able to: cry loud, have a pink color to the skin, breathe evenly and have a respiratory rate of 40 – 60 breaths per minute. what is the first thing you would do in the care of a baby that does not have these signs shortly after birth?**

**a) Dry baby with cloth,**

**b)** Use bag and face mask to help baby with respiration,

**c)** Suction of nose and mouth if necessary,

**d)** Slapping baby,

**3. What can be done to prevent premature LBW newborn baby from bleeding?**

**a)** Breastfeeding the child,

**b)** Not necessary to give any drugs,

**c)** Give Vitamin K,

**d) Give Vitamin K_1_,**

**4. Do you know what dose of Vitamin K_1_ to give to a Low birth weight baby according to recommendations in the National Guidelines?**

**a) 0.5 mg,** **b)** 1 mg, **c)** 2 mg, **d)** 5 mg,

**Infection management**

**5. A premature LBW newborn child can get eye infections after delivery. Which of the following would you use to prevent this from occurring?**

**a)** Do not apply anything,

**b)** Apply breast milk in the babies’ eyes,

**c)** Clean eyes with sterile water,

**d) Apply tetracycline eye ointment after cleaning eyes,**

**6. Taking care of the umbilical cord of a premature LBW newborn after delivery is important. Which of the following alternatives would you consider as important?**

**a)** Always clean your hands before touching the cord,

**b) Cut the cord with a clean instrument (for example, a razor blade),**

**c)** Use any sharp instrument for cutting the cord,

**d)** After cutting the cord, apply traditional herbs/medicines,

**7. What approach would you use to handle an umbilical cord that has any of the following signs: bad smell, oozing blood, small rashes around the umbilical area?**

**a)** Leave to dry,

**b)** Clean with water and soap,

**c) Clean with iodine solution,**

**d)** Apply antibiotic powder,

**Special care and monitoring**

**8. What is, in your opinion, the best way to stabilize the temperature of a premature LBW newborn baby?**

**a)** Bathing the baby in water of appropriate temperature,

**b)** By putting on clothes and cover head,

**c) Having the baby skin-to-skin with her/his mother,**

**d)** Keep the baby in a room with a temperature of 28-30°C,

**9. What action is important when taking care of a premature low birth weight baby immediately after birth?**

**a)** Bath the baby often,

**b)** Start breastfeeding early and frequently,

**c) Keep the child warm,**

**d)** Prevent infection from developing,

**10. How frequent should low birth weight babies be fed when weight is between 1250g-2500g**

**a)** Every 2 hours

**b) Every 3 hours**

**c)** Every 4 hours

**d)** Every 1 hours

**11. How frequent should low birth weight babies be fed when weight is less than 1250g**

**a) Every 2 hours**

**b)** Every 3 hours

**c)** Every 4 hours

**d)** Every 1 hours

**12. How frequent should the temperature be taken in 1500-2500 g babies in the first 24 hours**

**a) Every hour in the first 8hrs then every 12hrs**

**b**) Every 6hrs then every 12hrs

**c)** Every 12hrs then every 24hrs

**d)** Every 2hrs then every 4hrs

**13. How frequent should the temperature be taken in newborns below 1500 in the first 24hrs**

**a)** Every hour in the first 8hrs then every 12hrs

**b)** Every 6hrs then every 12hrs

**c) Every 30 min for 4hrs then hourly for 24hrs.**

**d)** Every 2hrs then every 4hrs

**14. How often should the respiratory rate be taken**

**a) Every 4hrs**

**b)** Every 6hrs

**c)** Every 12hrs

**d)** Every 2hrs

**15. How frequent should the oxygen saturations be taken?**

**a) Every 4hrs**

**b)** Every 6hrs

**c)** Every 12hrs

**d)** Every 2hrs

**16. How often should the heart rate be counted**

**a) Every 4hrs**

**b)** Every 6hrs

**c)** Every 12hrs

**d)** Every 2hrs

**17. How often should the blood glucose be taken?**

**a) 2hrs within delivery then 6hrly until infant getting enough feeds**

**b)** 30mins within delivery then 12hrly until infant getting enough feeds

**c)** 4hrs within delivery then 24hrs until infant getting enough feeds

**d)** 1hr within delivery then 4hrly until infant is getting enough feeds

**18. What is the acceptable daily weight gain for a premature?**

**a)** More than 10g per day

**b) More than 15g per day**

**c)** More than 25g per day

**d)** More than 30g per day

**19. A premature LBW is considered to have hypoglycemia when RBG is ………….and should be given a bolus of………….?**

**a)** Less than 3.3mmol/l, 5ms/kg of D10 IV stat

**b)** Less than 2.6mmol/l, 5ms/kg of D10 IV stat

**c)** Less than 3.3mmol/l, 2ms/kg of D10 IV stat

**d) Less than 2.6mmol/l, 2ms/kg of D10 IV stat**

**20. A LBW premature admitted to your unit with fast breathing >60breaths/minute, sever chest wall in drawing, grunting and cyanosis. what is the best management for this child?**

**a)** Put the preterm on oxygen via nasal prone

**b)** Put the preterm on oxygen via face mask

**c) Put the preterm on CPAP**

**d)** Put the preterm on ventilation
