# Supplementary material for: Nurses’ knowledge, perceived challenges, and recommended solutions regarding premature infant care: A mixed method study in the referral and tertiary hospitals in Dar es salaam, Tanzania
Source: PLoS One. 2023 Mar 29;18(3):e0281200. doi: 10.1371/journal.pone.0281200 (PMC10057798; doi:10.1371/journal.pone.0281200)
Supplement: S2 File — (DOCX) [file pone.0281200.s002.docx]

**Supportive Information 2(S2): Interview guide questions**

1. Can you describe the challenges you experience when providing care to premature newborns?
2. When you think about your own learning needs, what are the top 3 that you believe is most important?
3. When you consider learning needs in your workplace (unit as a whole), what do you believe is most important?
4. Can you describe what education was available for you as you entered the NICU and what ongoing education is available? For example, a scheduled orientation and ongoing professional development are scheduled regularly. Are these educational opportunities paid for as part of your position or volunteer time?
5. Can you describe or give examples of the educational opportunities offered at your institution after you started working there? Are these opportunities generally for nurses alone or in a multidisciplinary team environment?
6. There are many types of information that we need to care for ill and at-risk newborns. When you are seeking different types of information where do you look?
7. Is there a certain person (people) that you can go to within your hospital to help you find information?
8. Can you describe one or two barriers that you feel make it difficult to meet these priorities?
9. Can you describe how physicians and administration support the need for continuing professional development in your unit? How could his be improved?
10. If you could change one thing to improve your ability to provide care, what would it be?
