# Supplementary material for: Nurses’ knowledge, perceived challenges, and recommended solutions regarding premature infant care: A mixed method study in the referral and tertiary hospitals in Dar es salaam, Tanzania
Source: PLoS One. 2023 Mar 29;18(3):e0281200. doi: 10.1371/journal.pone.0281200 (PMC10057798; doi:10.1371/journal.pone.0281200)
Supplement: Attachment — Submitted filename: PONE-D-21-38982_reviewed.pdf [file pone.0281200.s004.pdf]

PLOS ONE

Nurses’ knowledge, challenges and solutions on the care of premature infants: a mixed method study in the referral and tertiary hospitals in Dar es salaam

--Manuscript Draft--

Manuscript Number:	PONE-D-21-38982
Article Type:	Research Article
Full Title:	Nurses’ knowledge, challenges and solutions on the care of premature infants: a mixed method study in the referral and tertiary hospitals in Dar es salaam
Short Title:	Nurses' knowledge ,challenges and solution on the care of premature infants in Dar es salaam hospitals
Corresponding Author:	Mwajuma Mwikali Mwamtenda, M.D ,Mmed<br>Muhimbili University College of Health Sciences: Muhimbili University of Health and Allied Sciences<br>Mombasa, Mombasa KENYA
Keywords:	
Abstract:	Background There has been an increase in preterm birth of about 2% in a span of 14 years (20002014) mainly from Asia and Sub-Saharan Africa. Nursing care is very crucial in these vulnerable infants and a lack of knowledge of health care providers is a contributing factor to morbidity and mortality. With the increase of preterm births, nurses’ knowledge adequacy, challenges and solutions on their care needs to be affirmed. Objective Aim of the study was to assess the nurses’ knowledge, its challenges and solutions on the care of premature infants. Material and Methodology A cross sectional study was conducted to determine adequacy of nurses’ knowledge on the care of premature infants and phenomenological study design using focus group discussions explored on the perceived challenges and solutions in acquiring on job training. Adequacy of knowledge was set at correct response of 50% or more on each domain. Quantitative data were analyzed using SPSS Version 23 and qualitative data were thematically categorized and themes derived. Results 55.8% of the nurses had never received on job training. Adequate knowledge among nurses was 94% in essential newborn care, 80.8% in infection prevention and management and 36.5% on special care and monitoring. Being more than 41 years old, a female nurse and working in the neonatal unit for at least 1-3 years were more likely to determine adequacy of knowledge in infection prevention and management. The main challenge in acquiring knowledge was lack of scheduled on job training. Solution recommended was to increase number of trainings. Conclusion Most nurses had not attended training on the care of premature infant. Special care and monitoring was most poorly performed. One of the major challenge faced is not being able to attend training due to poor scheduling and the solution to this is have frequent trainings Recommendations: Training should be conducted on special care and monitoring of premature infants. Nurses who were female, older than 41 years and those with 1 to 3 years of experience should be considered when planning for mentorship program on infection prevention and management.
Order of Authors:	Mwajuma Mwikali Mwamtenda, M.D ,Mmed
	Nahya Salim, MD, Mmed, phd
	Isabella Sylvester, MD, Mmed
	Emmanuel Munubhi, MD, Mmed
Additional Information:	
Question	Response
**Financial Disclosure**<br><br>Enter a financial disclosure statement that describes the sources of funding for the	the authors received no specific funding for this work

Powered by Editorial Manager® and ProduXion Manager® from Aries Systems Corporation

work included in this submission. Review the submission guidelines for detailed requirements. View published research articles from *PLOS ONE* for specific examples.<br><br>This statement is required for submission and **will appear in the published article** if the submission is accepted. Please make sure it is accurate.<br><br>**Unfunded studies**<br>Enter: *The author(s) received no specific funding for this work.*<br><br>**Funded studies**<br>Enter a statement with the following details:<br>• Initials of the authors who received each award<br>• Grant numbers awarded to each author<br>• The full name of each funder<br>• URL of each funder website<br>• Did the sponsors or funders play any role in the study design, data collection and analysis, decision to publish, or preparation of the manuscript?<br>• **NO** - Include this sentence at the end of your statement: *The funders had no role in study design, data collection and analysis, decision to publish, or preparation of the manuscript.*<br>• **YES** - Specify the role(s) played.<br><br>* typeset	
**Competing Interests**<br><br>Use the instructions below to enter a competing interest statement for this submission. On behalf of all authors, disclose any competing interests that could be perceived to bias this work—acknowledging all financial support and any other relevant financial or non-financial competing interests.<br><br>This statement is required for submission and **will appear in the published article** if the submission is accepted. Please make sure it is accurate and that any funding sources listed in your Funding Information	the author have declared that no competing interest exist

later in the submission form are also declared in your Financial Disclosure statement.<br><br>View published research articles from *PLOS ONE* for specific examples.<br><br>**NO authors have competing interests**<br><br>Enter: *The authors have declared that no competing interests exist.*<br><br>**Authors with competing interests**<br><br>Enter competing interest details beginning with this statement:<br><br>*I have read the journal's policy and the authors of this manuscript have the following competing interests: [insert competing interests here]*<br><br>* typeset	
**Ethics Statement**<br><br>Enter an ethics statement for this submission. This statement is required if the study involved:<br><br>• Human participants<br>• Human specimens or tissue<br>• Vertebrate animals or cephalopods<br>• Vertebrate embryos or tissues<br>• Field research<br><br>Write "N/A" if the submission does not require an ethics statement.<br><br>General guidance is provided below. Consult the submission guidelines for detailed instructions. **Make sure that all information entered here is included in the Methods section of the manuscript.**	Muhimbili University of Health and Allied sciences Institutional Review Board approved the study.<br>Approval number is MUHAS-REC-06-2020-307<br>form of consent obtained was written

**Format for specific study types**<br><br>**Human Subject Research (involving human participants and/or tissue)**<br>• Give the name of the institutional review board or ethics committee that approved the study<br>• Include the approval number and/or a statement indicating approval of this research<br>• Indicate the form of consent obtained (written/oral) or the reason that consent was not obtained (e.g. the data were analyzed anonymously)<br><br>**Animal Research (involving vertebrate animals, embryos or tissues)**<br>• Provide the name of the Institutional Animal Care and Use Committee (IACUC) or other relevant ethics board that reviewed the study protocol, and indicate whether they approved this research or granted a formal waiver of ethical approval<br>• Include an approval number if one was obtained<br>• If the study involved *non-human primates*, add *additional details* about animal welfare and steps taken to ameliorate suffering<br>• If anesthesia, euthanasia, or any kind of animal sacrifice is part of the study, include briefly which substances and/or methods were applied<br><br>**Field Research**<br><br>Include the following details if this study involves the collection of plant, animal, or other materials from a natural setting:<br>• Field permit number<br>• Name of the institution or relevant body that granted permission	
**Data Availability**<br><br>Authors are required to make all data underlying the findings described fully available, without restriction, and from the time of publication. PLOS allows rare exceptions to address legal and ethical concerns. See the PLOS Data Policy and FAQ for detailed information.	Yes - all data are fully available without restriction

A Data Availability Statement describing where the data can be found is required at submission. Your answers to this question constitute the Data Availability Statement and **will be published in the article**, if accepted.<br><br>**Important:** Stating 'data available on request from the author' is not sufficient. If your data are only available upon request, select 'No' for the first question and explain your exceptional situation in the text box.<br><br>Do the authors confirm that all data underlying the findings described in their manuscript are fully available without restriction?	
**Describe where the data may be found in full sentences. If you are copying our sample text, replace any instances of XXX with the appropriate details.**<br><br>• If the data are **held or will be held in a public repository**, include URLs, accession numbers or DOIs. If this information will only be available after acceptance, indicate this by ticking the box below. For example: *All XXX files are available from the XXX database (accession number(s) XXX, XXX.*).<br>• If the data are all contained **within the manuscript and/or Supporting Information files**, enter the following: *All relevant data are within the manuscript and its Supporting Information files.*<br>• If neither of these applies but you are able to provide **details of access elsewhere**, with or without limitations, please do so. For example:<br><br>*Data cannot be shared publicly because of [XXX]. Data are available from the XXX Institutional Data Access / Ethics Committee (contact via XXX) for researchers who meet the criteria for access to confidential data.*<br><br>*The data underlying the results presented in the study are available from (include the name of the third party*	all relevant data are within the manuscript and its supporting information files

Powered by Editorial Manager® and ProduXion Manager® from Aries Systems Corporation

and contact information or URL).<br>• This text is appropriate if the data are owned by a third party and authors do not have permission to share the data.<br><br>* typeset	
Additional data availability information:	Tick here if your circumstances are not covered by the questions above and you need the journal’s help to make your data available.

Manuscript

Click here to access/download;Manuscript;Mwajumas manuscript.docx

Nurses' knowledge, challeng[illegible]nd solutions on the care of premature infants: a mixed method study in the referral and tertiary hospitals in Dar es salaam

Mwajuma Mwikali [1, #a, *], Nahya Salim [1*], Isabella Sylvester [2], Emmanuel Munubhi [1]

1 Department of Peadiatrics, School of Medicine, Muhimbili University of Health and Allied Sciences (MUHAS), Dar es Salaam, Tanzania

2 Department of Peadiatric Muhimbili National Hospital, Dar es Salaam, Tanzania

#a Current address: Department of Peadiatrics Msambweni County Referral Hospital (MCRH), Kwale, Kenya

* Corresponding authors

E-mail:Bejummer@gmail.com (MM)

E-mail: nsalim@ihi.or.tz (NS)

MM and NS are joint authors.

Abstract

Background: There has been an increase in preterm birth of about 2% in a span of 14 years (2000-2014) mainly from Asia and Sub-Saharan Africa. Nursing care is very crucial in these vulnerable infants and a lack of knowledge of health care providers is a contributing factor to morbidity and mortality. With the increase of preterm births, nurses' knowledge adequacy, challenges and solutions on their care needs to be affirmed.

Objective**:** Aim of the study was to assess the nurses' knowledge, its challenges and solutions on the care of premature infants.

Material and Methodology: A cross sectional study was conducted to determine adequacy of nurses' knowledge on the care of premature infants and phenomenological study design using focus group discussions explored on the perceived challenges and solutions in acquiring on job training. Adequacy of knowledge was set at correct response of 50% or more on each domain. Quantitative data were analyzed using SPSS Version 23 and qualitative data were thematically categorized and themes derived.

Results 55.8% of the nurses had never received on job training. Adequate knowledge among nurses was 94% in essential newborn care, 80.8% in infection prevention and management and 36.5% on special care and monitoring. Being more than 41 years old, a female nurse and working in the neonatal unit for at least 1-3 years were more likely to determine adequacy of knowledge in infection prevention and management. The main challenge in acquiring knowledge was lack of scheduled on job training. Solution recommended was to increase number of trainings.

Conclusion: Most nurses had not attended training on the care of premature infant. Special care and monitoring was most poorly performed. One of the major challenge faced is not being able to attend training due to poor scheduling and the solution to this is have frequent trainings

Recommendations: Training should be conducted on special care and monitoring of premature infants. Nurses who were female, older than 41 years and those with 1 to 3 years of experience should be considered when planning for mentorship program on infection prevention and management.

Introduction

Premature infants are prone to complications like respiratory distress syndrome and infection. These are one of the main causes of mortality among these infants (1).

Approximately 1 million out of 15 million born prematurely globally die each year due to complications associated with preterm birth (2)

With preterm birth rates on the rise (2) and according to every preemie scale of 2019, in Tanzania approximately 336,000 preterms are born in a year. 3.4% of them die due to their complications.

From low-resource settings one of the factors that contribute to Neonatal Mortality is inadequate training & low staff competence (3)

Some of the factors linked to inadequate care provided is health worker shortages which is compounded by a lack of specific knowledge and competencies (4)

In Northern Tanzania, prematurity was the most common cause of mortality in low birth weight infants (5) but causes of these mortality was not determined including quality of care.

There is a link between adequate care provided in relation to adequate knowledge among health care providers. Nurses are the backbone care providers for these infants their knowledge needs to be ascertained.

The main aim of this study was to assess nurses' knowledge on the care of premature infants in 3

main domains, essential newborn care, infection prevention and management and special care and monitoring. This study also aimed at Determining factors associated with their knowledge.

Further exploring their challenges and solutions in acquiring this knowledge was of importance.

Methodology

A mixed-method was used combining both descriptive cross-sectional study with qualitative phenomenological study design. The quantitative component was determining the nurses' knowledge and associated factors whereas the qualitative component was an additive to the study to determine general experience, challenges, and solutions in acquiring knowledge on premature infant care among the neonatal unit nurses. This study was conducted in the Dar es Salaam region, the largest city in Tanzania with a population of 6.7 million. Dar es Salaam has five districts including kinondoni, Ilala, Temeke, Ubungo, and Kigamboni. There are three regional referral hospitals (RRH) namely Amana, Mwananyamala and Temeke. All the three RRHs refer newborns to two tertiary hospitals, Muhimbili National Hospital (MNH), Upanga, and Mloganzila branch. This study was conducted in the three RRHs and one of the tertiary branches, MNH- Upanga. MNH-Upanga alone accounts for approximately 369 preterm deliveries per month not accounting for the referral ins (19) with bed capacity of approximately 100 in the specific premature ward. In the regional referral hospitals, preterm deliveries can be approximately 42 per month in all the hospitals (20) with a bed capacity of approximately 15 to 20 which is for both term and preterm newborns. Premature newborns weighing less than 1.8kg and those that are sick irrespective of their weight are admitted to the neonatal unit. Each facility has a neonatal intensive care unit (NICU) that offers special service and care for these premature infants. Since both facility levels provide newborn care, it gave room for comparability in terms of the level of knowledge.

Study population were all nurses who worked in the neonatal unit in these hospitals and those that specifically took part in caring for these premature infants.

The study was conducted between September 2020 to January 2021

~~Quantitative study design~~

Sample size was acquired using kish leslie formula with 69% proportion of nurses with adequate knowledge on preterm care then using formula for finite population using population size we got 52.

Those excluded from the study are nurses who declined consent to participate.

Data was collected using a self-administered questionnaire (S1) with the tool adopted from a study done in Masindi Uganda (6) which later modified using the Tanzanian Neonatal national guideline of 2019. A pilot was done and questionnaire corrected.

With the assistance of a selected nurse in each respective hospital, consecutive sampling was done until sample size was attained.

Data was entered and cleaned in SPSS Version 23. The dependent variables were knowledge on three main domains: 1. Essential newborn care 2. Infection prevention and management and 3. Special care and monitoring. The independent variables were sociodemographic factors (age, gender, cadre), institutional factors (either working in the regional hospital or the national hospital), level of education, years of experience, and when was the last training/workshops on preterm care attended. Frequency tables were generated to determine the social demographic distribution of the nurses. Ranges were generated in the variables of age and years of experience.

The questions were computed and each question was graded as 1 and 0 and dichotomized as correct and incorrect responses respectively. For each of the themes, nurses were judged to have adequate knowledge if they responded correctly more than 50% of the questions in each domain.

Chi-square or Fischer's test p-value was used for the categorical variables against adequate and inadequate knowledge in each domain to determine association factors and any p-value of < 0.05 in logistic regression was determined as statistically significant in the association of nurses' knowledge in the care of preterm newborns.

~~Qualitative study design~~

Focus group discussions (FGD) which were done after the completion of the quantitative data collection. Nurses eligible for the FGD were those with experience of a year and more in the neonatal unit caring for premature infants. Purposive-homogenous sampling was done. The number of FGD was held based on saturation attainment. The aim was to conduct FGD in both the national hospital and regional hospital in order to get their experiences based on the 2 different levels of hospital. From the regional hospital, the hospital with most nurses with inadequate knowledge in special care and monitoring was selected. Two FDG were done, one was conducted in Temeke regional referral hospital and the other at MNH, Upanga with a total of 6 participants in each FGD.

Interview guide was adapted from a study by Campbell et al (7) then modified to suit the objectives of this study (S2)

Recordings were transcribed and with the assistance of an expert in qualitative data analysis, the transcribed notes were read several times to determine codes using the codebook created. The

codes were written in MS Excel then made into categories, subthemes, and themes. Themes derived included general experience in acquiring knowledge, challenges perceived, and solutions to perceived challenges.

Results

Quantitative

A total of 52 nurses were enrolled.9 (17%) were from Amana, Mwananyamala 8 (15%), Temeke 10 (19%), and the MNH, Upanga 25 (48.1%). Almost 85% (44/52) of the nurses were female.At the time of the study, 56% (29/52) of the nurses had never had the privilege to attend training on the care of premature infants.(Fig 1)

Fig 1. social demographic characteristics

In essential newborn care, overall all hospitals had a poor response on HBB question compared to other questions in the same domain. In infection prevention and management, overall umbilical cord care response was poor compared to other questions in this domain. (Fig 2)

Fig 2. Nurses' knowledge on Essential New Born Care (ENC) and Infection Prevention and Management (IPM) domain by hospitals.

Knowledge on special care and monitoring provided to the premature infants was poorly responded in the following questions; 19% knew how to do blood glucose monitoring within the first 24 hours after delivery, temperature monitoring in the preterm newborn with less than 1500 g weight with 25% of the nurses having a correct response and Only 29% of the nurses knew the acceptable daily weight gain. (Fig 3)

F[illegible]3. Nurses' knowledge on Special Care and Monitoring domains in all hospitals

Factors associated with nurses' knowledge

94.2% (49/52) of the nurses had adequate knowledge on essential newborn while 80.8% (42/52) and only 36.5%(19/52) had adequate knowledge on infection prevention and management and special care and monitoring respectively. There was no statistical significance association between adequacy in knowledge in different domains with the different independent variables.

93.3%(14/15) of the nurses who were more than 41 years of age had adequate knowledge on infection prevention and management (IPM) with adjusted odds ratio (AOR) of 2.3 and 95% confidence interval (CI) 0.11-48.1. Nurses who were between 31 and 40 years old, 87.5% (14/16) had adequate knowledge on IPM with AOR of 1.5 and 95% CI 0.18-12.14.

From the regional referral hospitals, 88.9% (24/27) of the nurses had adequate knowledge on IPM where as 72% (18/25) of nurses from MNH had adequate knowledge with AOR 0.35 and 95% CI 0.04-2.61.

From the female nurses, 86.4% (38/44) had adequate knowledge in IPM with AOR 3.5 and 95% CI 0.60-20.7.

All the nurses who have worked in neonatal unit for more than 6 years had adequate knowledge on IPM while those who had worked between 1 to 3 years, 82.6%(19/23) had adequate knowledge with AOR of 2.3 and 95% CI 0.3-14.28 and those that worked between 4 to 6 years, 83.3% (5/6) had adequate knowledge with AOR 1.18 and 95% CI 0.04-33.08.(Table 1)

Table 1: Factors associated with nurse's knowledge

Domains		ENC		IPM				SCM
	**Adequate Frequency**	**Inadequate Frequency**	**Adequate Frequency**	**Inadequate Frequency**	**COR (95% CI)**	**AOR (95% CI)**	**Adequate Frequency**	**Inadequate Frequency**
**Variables**	**49(94.2%)**	**3(5.8%)**	**42(80.8%)**	**10(19.2%)**			**19(36.5%)**	**33(63.5%)**
**Age groups**								
21-30 yrs.	19(90.5)	2(9.5)	14(66.7)	7(33.3)	1	1	6(28.6)	15(71.4)
31-40 yrs.	15(93.8)	1(6.2)	14(87.5)	2(12.5)	3.5(0.62-19.88)	1.5(0.18-12.94)	5(31.2)	11(68.8)
> 41 yrs.	15(100)	0	14(93.3)	1(6.7)	7(0.75-64.61)	2.3(0.11-48.10)	8(53.3)	7(46.7)
**Hospital Levels**								
RRH	26(96.3)	1(3.7)	24(88.9)	3(11.1)	1	1	9(33.3)	18(66.7)
MNH	23(92.0)	2(8)	18(72)	7(28)	0.32(0.07 – 1.41)	0.35(0.04 – 2.61)	10(40)	15(60)
**Gender**								
Male	8(100)	0	4(50)	4(50)	1	1	3(37.5)	5(62.5)
Female	41(93.2)	3(6.8)	38(86.4)	6(13.6)	6.33(1.23-32.37)	3.5(0.60-20.70)	16(36.4)	28(63.6)
**Cadre**								
EN	16(100)	0	15(93.7)	1(6.3)			7(43.7)	9(56.3)
ANO	23(95.8)	1(4.2)	17(70.8)	7(29.2)			8(33.3)	16(66.7)
NO	10(83.3)	2(16.7)	10(83.3)	2(16.7)			4(33.3)	8(66.7)
**Years of service**								
< 1 yr.	11(91.7)	1(8.3)	7(58.3)	5(41.7)	1	1	3(25)	9(75)
1- 3 yrs.	21(91.3)	2(8.7)	19(82.6)	4(17.4)	3.39(0.70-16.38)	2.3(0.36-14.28)	7(30.4)	16(69.6)
4- 6 yrs.	6(100)	0	5(83.3)	1(16.7)	3.57(0.31-40.75)	1.18(0.04-33.08)	3(50)	3(50)
> 6 yrs.	11(100)	0	11(100)	0	1		6(54.5)	5(45.5)
**Training attended**								
not attended	27(93.1)	2(6.9)	23(79.3)	6(20.7)			9(31.1)	20(68.9)

attended < 1 yr. ago	11(91.7)	1(8.3)	10(83.3)	2(16.7)	5(41.7)	7(58.3)
attended > 1 yr. ago	11(100)	0	9(81.8)	2(18.2)	5(45.5)	6(54.5)
**Level of Education**						
certificate	16(100)	0	15(93.7)	1(6.3)	7(43.7)	9(56.3)
diploma	23(95.8)	1(4.2)	17(70.8)	7(29.2)	8(33.3)	16(66.7)
degree	10(83.3)	2(16.7)	10(83.3)	2(16.7)	4(33.3)	8(66.7)

AOR- Adjusted Odds Ratio, ANO-Assistant Nursing Officer, COR- Crudes Odds Ratio, EN- Enrolled Nurse, ENC-Essential Newborn Care, IPM-Infection Prevention and Management, MNH- Muhimbili National Hospital, NO- Nursing Officer, RRH- Regional Referral Hospital

Qualitative results

3 main themes emerged from the FGD

General experience in acquiring knowledge

The nurses felt that there is need to know more about how to use the equipment at work.

"…knowledge that we need most is how to operate the machines (i.e. concentrator) that are brought. We are not taught how to use them. They just show us the parts. The only thing we understand is when it doesn't bubble but we don't know how to operate them…" (P2 Temeke RRH)

Main source of knowledge is through learning from one another.

"…We usually hold CME whereby one is assigned a topic based on standard operating procedures (SOP) or guideline to teach each other in our departments…" (P2 MNH, Upanga)

Perceived challenges

While at work, both in the regional and national hospital, lack of equipment/ resources was common.

"…For instance, when a newborn is brought in sick you will find that one bed can have two or even three or even four babies…" (P2 Temeke RRH)

"…in these coming months of January and February we expect an increase in delivery of newborns and with the understaffing issue at hand, it becomes a big challenge …" (P1 MNH, Upanga)

While acquiring knowledge, the selection process was thought to be an obstacle.

"…When it comes to deciding who goes for training, it's the task of the in-charge nurse of the ward. But the issue isn't with just the in-charge nurse, the issue is the number of nurses that could be released to go for training…" (P2 MNH, Upanga)

Solutions to the perceived challenges

Those that are related to work, on job training for every newly employed staff was recommended.

"…Those who are new to the assigned ward either newly employed or just a volunteer or even a doctor, they should undergo an on job training on how to care for the preterms…" (P5 MNH, Upanga)

When it came to improving knowledge, learning from other nurses was advocated in both the regional and national hospital

"…Amongst the nurses at our workplaces can teach one another about something new they learned…"(P1 Temeke RRH)

Discussion

Essential Newborn care

In our study most of the nurses (94%) had adequate knowledge in essential newborn care unlike the study done in Masindi Uganda whereby only 41.9% of nurses had adequate knowledge (6). This would be explained by the fact that 45% of the nurses, which was the majority number from the study in Uganda, were mainly those from outpatient and did not do daily inpatient care for newborns. Overall in our study, there was low knowledge on step wise management of help baby breath (HBB) algorithm which was consistent in the study done in Uganda where 21.9% of the

nurses had the knowledge (6). In our study 55.8% of the nurses had also not had the privilege to attend any training on premature infant care due to the COVID pandemic prohibition of social gatherings. This would explain the low level of knowledge in HBB algorithm which is usually provided through training by CCBRT every six months. All newborns at birth need simple care which includes the help baby breath protocol which is sufficient for 80-90 % of the time when done appropriately.

Infection Prevention and Management

80.8% of the nurses had adequate knowledge on infection prevention and management in our study compared to the study from Uganda, 13.3% (6). Umbilical cord care by ensuring the use of a clean blade when cutting the cord as means of prevention of infection was the most poorly answered question in this domain. Whereas in the study done in Uganda this similar question had a better response with 72.1% having a correct response (6). While the study from Ethiopia, 61% of the nurses had a correct response (8). In a cross-sectional study with an observational component including 6 sub-Saharan African countries including Tanzania showed that 94% of the health care workers used a clean blade to cut the cord (9). Despite cord care having the poorest response, the practiced care is known by many health care workers. In our study the poor response might be explained by the fact that initial cord care, which was what the question was assessing, is provided by labor ward nurses and the midwife. In our study the nurses who participated were mainly neonatal nurses, who didn't practice daily initial cord care

Special Care and Monitoring

In our study, 36.5% of the nurses had adequate knowledge of special care and monitoring. While in a cross-sectional observational study done in Pakistan, 46%-54% of the nurses in the neonatal unit performed vital signs monitoring as required (10). This study didn't assess knowledge directly like in our study so comparability with our study is questionable. The results from our study would be explained by the fact that nursing staff ratio to number of infants is low. Regular and scheduled monitoring becomes a challenge when few nurses are available to provide care. Vital signs monitoring in neonates is the most integral part of their care whereby in modern medicine, continuous vital signs monitoring is being preferred instead of intermittent monitoring (11). It is known that trends in vital signs can predict some of the complications that the preterm newborns are at risk of such as sepsis, NEC, brain injury, BPD, and even mortality. This emphasizes the importance of constant monitoring of vital signs.

Among the poorly answered questions was the monitoring of blood glucose with 19.2% of the nurses having correct responses. This is very crucial in the management of premature infants because impaired glucose control in very preterm newborns has been associated with an increase in morbidity, poor neurologic outcome, and even mortality. This has enabled studies done to compare continuous glucose monitoring which was found to be better in terms of glucose control than intermittent monitoring (12). Findings in our study could be due to the inability to frequently monitor random blood glucose due to inadequate supply of strips. This limits the practice of frequent monitoring.

Monitoring of temperature in those premature infants with weight less than 1500g was not well known with only 25% of the nurses having the correct response. Lack of adequate nursing staff

might contribute to lack of proper monitoring. This might also be due to the availability of continuous temperature monitoring for premature infants placed on a servo machine. This might not be the case for every premature infant admitted to the neonatal unit. Only the high-risk premature infants are placed under the servo machine and every preterm irrespective of being high risk or not needs monitoring. Newborns who are at risk of hypothermia are those with very low weight and those with low gestational age at the time of birth. This has a detrimental effect on their neurodevelopment or even leads to death (13). The importance of proper temperature monitoring in these premature infants and the need to improve knowledge should be prioritized.

About 28.1% of the nurses knew acceptable daily weight gain of the premature infant despite being a daily routine practice done in the neonatal unit. Weight monitoring is crucial since it is an indicator of the energy intake and expenditure (internal heat loss through basal metabolism and physical activity) and enables one to decide on how to intervene when there isn't adequate weight gain i.e. the use of parenteral nutrition. Poor growth during admission in the neonatal unit has been linked to poor neurodevelopmental outcomes (14). So there is a need to emphasize the proper daily weight monitoring to ensure proper action is put in place.

Difference in knowledge among the nurses

Nurses who were older than 41 years of age were 2.3 times more likely to have adequate knowledge on infection prevention and management compared to those who were less than 30 years old. This was similar to a study done in Mosul whereby nurses aged 40-49 years had more knowledge on general care of premature infants compared to the younger nurses (15).

Nurses at the national level were 0.35 less likely to have adequate knowledge in infection management compared to those in regional referral hospitals. The study done in Uganda showed those who work on a higher level of the hospital were 1.1 times more likely to have adequate knowledge in infection management (6). This was inconsistent with our study where more nurses from the regional hospital had adequate knowledge. The nurses in the regional hospitals that participated in this study did care for both premature and term infants so they were aware of proper cord care which was the most poorly responded question in the national hospital. Prevalence of cord sepsis among premature infants is not known currently. This cannot be linked with the knowledge the nurses from the national hospital have in respect to cord care in premature infants. Females nurses were 3.2 times more likely to have adequate knowledge in infection prevention and management compared to male nurses. This could be explained by the high numbers of female nursing staff compared to the males.

Nurses that worked for 1-3 years were 2 times more likely to have adequate knowledge in infection prevention and management compared to those with more years of experience. This could also be explained by the fact that the nurses with less years of experience in neonatal ward were updated on current cord care available since they were recently from school.

Experience, challenges and solutions in acquiring knowledge among nurses

Based on the qualitative part of the study, the general experience in acquiring knowledge is based on their learning needs when caring for the premature infants and the sources of knowledge available for them. The learning needs stated by the nurses is knowing how to operate the

equipment at work and being up to date on the management of premature infants. Their main source of knowledge while at work was fellow nurses and doctors.

These findings were similar to those found in the Indian study (7). When considering about training, perhaps in house mentorship should be considered and strengthened in the neonatal ward. This will contribute to better care for these infants (18).

In our study, challenges that were perceived by the nurses were those related to work which included understaffing, work overload, and lack of equipment at work. This was consistent with the study done in India by Campbell et al. (7). These factors affect the overall care the preterm newborns receive.

Challenges perceived while acquiring knowledge included not having a standard selection process for those attending training, this being similar to a study by Campbell et al (7). In the quantitative data from this study most of the nurses had not attended any training on premature infant care and this could be explained by the poor selection process and lack of on job training plan.

As part of the solution to the challenges faced, it was suggested that senior nurses and doctors should be tasked to teach other nurses. Team work among health care workers has been seen as an integral part in providing care (16). In terms of challenges faced while working some of the solutions implicated were the availability of on job training for every new employee and to have a training schedule that will allow all nurses to attend. According to WHO's a new roadmap on human resources to ensure all newborns survive and thrive, on-the-job training has been suggested as one of the strategies for improvement (17). All these findings were similar to the study by Campbell et al. (7). This study was done before the implementation of the Newborn Essential Solutions and Technologies (NEST 360) program in Dar es Salam which is currently in place to

ensure training on how to manage newborns using the current technology not forgetting the basic care.

Strength of the study

Using quantitative data, we captured the inadequate knowledge in special care and monitoring while the added component of the qualitative part of the study was able to highlight challenges in acquiring knowledge and suggested solutions to these challenges.

Study limitations

1. In the public health center in Dar es Salam only the regional and national hospitals had neonatal intensive care units. The number of employed nurses working in the NICU from all 4 hospitals was approximately 54 at the time of the study. The sample size is too small with lack of a standard tool to assess knowledge, so enable the generalizability of the data becomes a challenge.
2. A study that has both cross sectional and observational component should be done based on assessing premature infant care to ascertain adequate care provided rather than making this assumption with only assessment of knowledge.
3. After FGD, transcripts were not returned to participants for comments and correction. The participants of the FGD were not able to provide feedback on the transcripts for verifying accuracy and clarification of results. There was a possibility of missed out information. Although the practice of verification of transcripts is still questionable and might not add any value to the data.

Conclusion

1. 55.8% of the nurses had not attended any training on premature infant care after employment in neonatal unit. Less than 50% of nurses from the regional referral hospitals knew the HBB algorithm management. Less than 50% of the nurses in all the hospitals knew proper cord care. More than 80% of the nurses had adequate knowledge on essential newborn care and infection prevention and management but only 37% of the nurses had adequate knowledge in special care and monitoring which is a vital part in the care of premature infants.
2. Being older than 41 years, female nurses and working in the neonatal unit for at least 1-3 years were more likely to determine adequate knowledge in infection prevention and management.
3. Nurses preferred to learn from one another and there was a need for updates on how to use the neonatal equipment and general care for the premature infants.
4. The main challenge faced, is not being able to attend training as frequently as expected. This was contributed by low staff numbers and suggested frequent training should be held to allow continuity on learning.

Recommendations

1. Continuous medical education should emphasize more on HBB algorithm management, cord care and the special care and monitoring of premature infants.
2. On job training plan should be in place to allow effective coverage on the knowledge to care for premature infants. Hospitals and Government commitment is needed to ensure effective implementation plans.

3. Mentorship should be utilized as a means of ensuring knowledge gain within and between hospitals.

Acknowledgments

[illegible] would like to thank the almighty for all He has provided for me thus far including the support of my amazing supervisors, the department of peadiatrics of MUHAS, the nurses who took part in my study and most of all my family.

Reference

1. Muhe LM, McClure EM, Nigussie AK, Mekasha A, Worku B, Worku A, Demtse A, Eshetu B, Tigabu Z, Gizaw MA, Workneh N. Major causes of death in preterm infants in selected hospitals in Ethiopia (SIP): a prospective, cross-sectional, observational study. The Lancet Global Health. 2019 Aug 1;7(8): e1130-8.
2. Liu L, Oza S, Hogan D, Chu Y, Perin J, Zhu J, Lawn JE, Cousens S, Mathers C, Black RE. Global, regional, and national causes of under-5 mortalities in 2000–15: an updated systematic analysis with implications for the Sustainable Development Goals. The Lancet. 2016 Dec 17;388(10063):3027-35.
3. Jalil F. Perinatal health in Pakistan: a review of the current situation. Acta Paediatrica. 2004 Oct;93(10):1273-9.
4. Moxon SG, Lawn JE, Dickson KE, Simen-Kapeu A, Gupta G, Deorari A, Singhal N, New K, Kenner C, Bhutani V, Kumar R. Inpatient care of small and sick newborns: a multi-

country analysis of health system bottlenecks and potential solutions. BMC pregnancy and childbirth. 2015 Dec;15(2):1-9.

5. Mmbaga BT, Lie RT, Olomi R, Mahande MJ, Kvåle G, Daltveit AK. Cause-specific neonatal mortality in a neonatal care unit in Northern Tanzania: a registry based cohort study. BMC pediatrics. 2012 Dec;12(1):1-0.
6. Ayiasi RM, Criel B, Orach CG, Nabiwemba E, Kolsteren P. Primary healthcare worker knowledge related to prenatal and immediate newborn care: a cross sectional study in Masindi, Uganda. BMC health services research. 2014 Dec;14(1):1-1.
7. Campbell- Yeo M, Deorari A, McMillan DD, Singhal N, Vatsa M, Aylward D, Scotland J, Kumar P, Joshi M, Kalyan G, Dol J. Educational barriers of nurses caring for sick and at- risk infants in I ndia. International nursing review. 2014 Sep;61(3):398-405.
8. Abdu H, Gebrselassie M, Abdu M, Mare KU, Tadesse W, Liben ML. Knowledge and practice of immediate newborn care among midwives and nurses in public health facilities of Afar regional state, Northeast Ethiopia. BMC pregnancy and childbirth. 2019 Dec;19(1):1-0.
9. De Graft-Johnson J, Vesel L, Rosen HE, Rawlins B, Abwao S, Mazia G, Bozsa R, Mwebesa W, Khadka N, Kamunya R, Getachew A. Cross-sectional observational assessment of quality of newborn care immediately after birth in health facilities across six sub-Saharan African countries. BMJ open. 2017 Mar 1;7(3).
10. RAFIQUE R, HUSSAIN M, AFZAL M, GILANI SA. Assessment of Nursing Care Skills in Neonatal Unit: A Cross Sectional Observational Study. Pakistan Journal Of Neurological Surgery. 2020 Jul 14;24(2):187-94.

11. Kumar N, Akangire G, Sullivan B, Fairchild K, Sampath V. Continuous vital sign analysis for predicting and preventing neonatal diseases in the twenty-first century: big data to the forefront. Pediatric research. 2020 Jan;87(2):210-20.

12. Galderisi A, Facchinetti A, Steil GM, Ortiz-Rubio P, Cavallin F, Tamborlane WV, Baraldi E, Cobelli C, Trevisanuto D. Continuous glucose monitoring in very preterm infants: a randomized controlled trial. Pediatrics. 2017 Oct 1;140(4).

13. Ting JY, Synnes AR, Lee SK, Shah PS. Association of admission temperature and death or adverse neurodevelopmental outcomes in extremely low-gestational age neonates. Journal of Perinatology. 2018 Jul;38(7):844-9

14. Bocca-Tjeertes IF, Reijneveld SA, Kerstjens JM, De Winter AF, Bos AF. Growth in small-for-gestational-age preterm-born children from 0 to 4 years: the role of both prematurity and SGA status. Neonatology. 2013;103(4):293-9.

15. Mohammed QN, Alsawaf BF. Assessment of Nursing staff's Knowledge and Practice regarding Care of Premature Babies in Mosul Teaching Hospitals. Mosul Journal of Nursing. 2016 Jun 1;4(2):70-3.

16. Baker U, Hassan F, Hanson C, Manzi F, Marchant T, Swartling Peterson S, et al. Unpredictability dictates quality of maternal and newborn care provision in rural Tanzania-A qualitative study of health workers' perspectives. BMC Pregnancy Childbirth. 2017;17(1):55.

17. World Health Organization. Roadmap on human resource strategies to improve newborn care in health facilities in low-and middle-income countries.

18. Baker U, Petro A, Marchant T, Peterson S, Manzi F, Bergstrom A, et al. Health workers' experiences of collaborative quality improvement for maternal and newborn care in rural

Tanzanian health facilities: A process evaluation using the integrated 'Promoting Action on Research Implementation in Health Services' framework. PLoS One. 2018;13(12):e0209092.

19. Rugumisa BT, Bongcam- Rudloff E, Lukumay MS, Lyantagaye SL. Factors associated with risk of preterm delivery in Tanzania: A case- control study at Muhimbili National Hospital. International Journal of Gynecology & Obstetrics. 2021 Aug;154(2):318-23.
20. Mahapula FA, Kumpuni K, Mlay JP, Mrema TF. Risk factors associated with pre-term birth in Dar es Salaam, Tanzania: a case-control study. Tanzania Journal of Health Research. 2016;18(1)

Supporting information

S1 File. Self-administered questionnaire

S2 File. Interview guide questions

Fig 1. social demographic characteristics

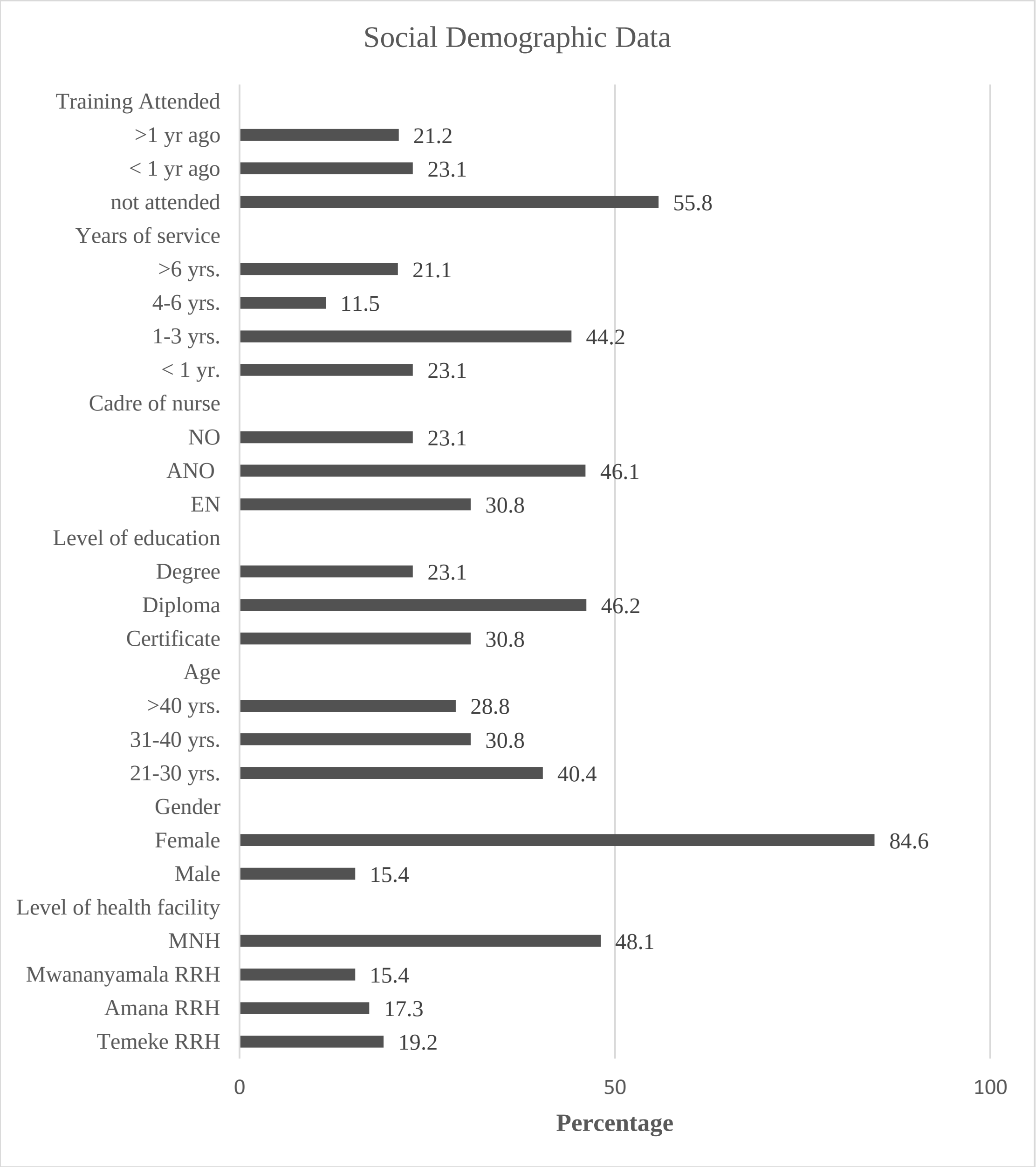

Fig 2. Nurses' knowledge on Essential New Born Care (ENC) and Infection Prevention and Management (IPM) domain by hospitals.

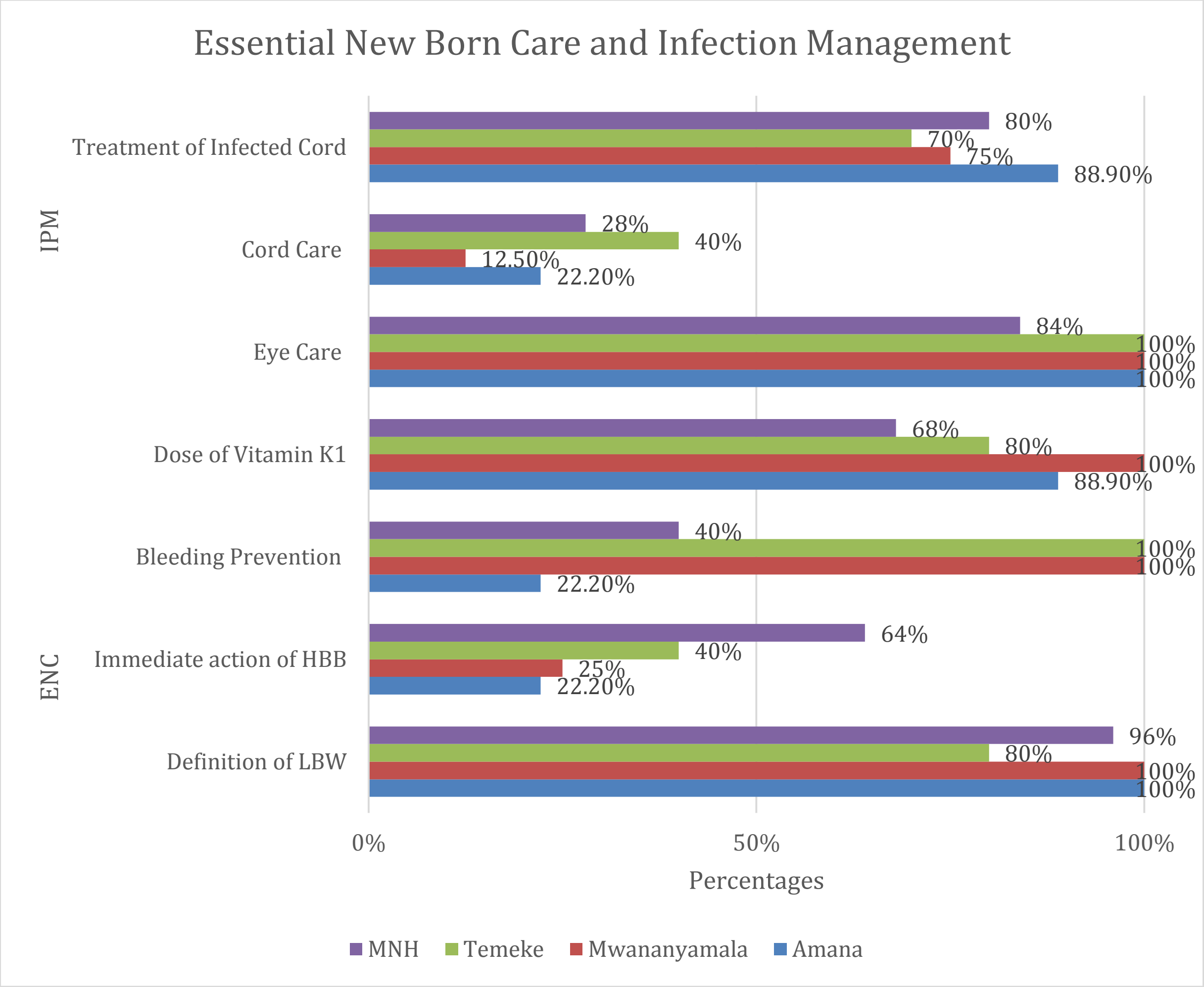

HBB-help baby breath, LBW-low birth weight

Fig 3. Nurses' knowledge on Special Care and Monitoring domains in all hospitals

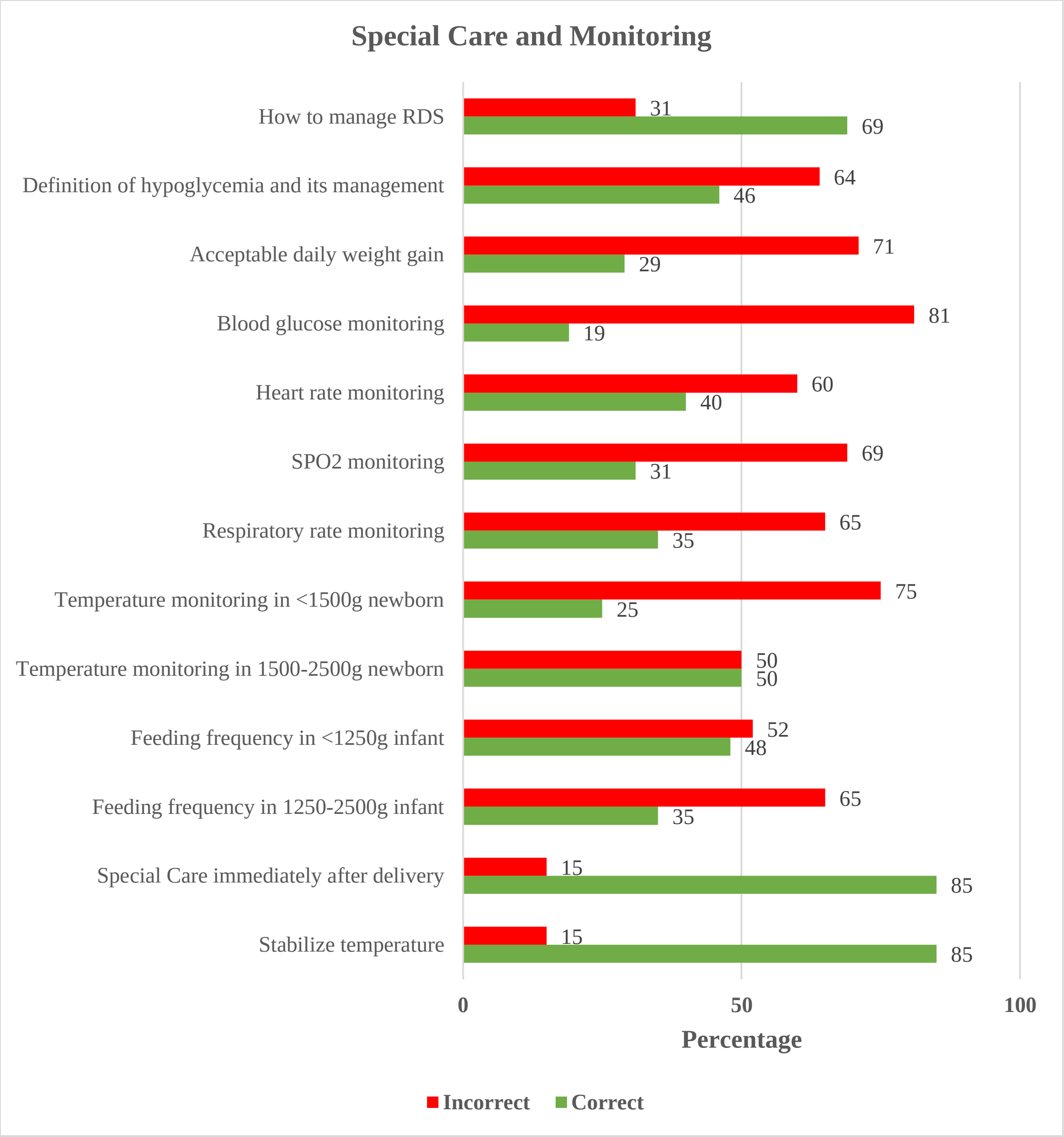

RDS- respiratory distress syndrome

Supportive Information 1(S1): Self-administered questionnaire English version
Questionnaire #................Name of your facility……………………………………………

Circle and fill in appropriately

Gender:

Male

Female

Age:

Cadre of health worker:

Highest level of education you hold?

Certificate

Diploma

degree

How many years have you served as a neonatal nurse in caring for the premature LBW babies? (Completed years)

When did you last attend a training or workshop on premature LBW newborn care?

<u>**Essential Newborn Care**</u>

1. What is the definition of low birth weight for newborn child?

a) Weight is less than 3000 grams,

b) Weight is less than 2500 grams,

c) Weight is less than 1500 grams,

d) Weight is less than 1000 grams,

2. Shortly after birth a premature LBW newborn baby should be able to: cry loud, have a pink color to the skin, breathe evenly and have a respiratory rate of 40 – 60 breaths per minute. what is the first thing you would do in the care of a baby that does not have these signs shortly after birth?

a) Dry baby with cloth,

b) Use bag and face mask to help baby with respiration,

c) Suction of nose and mouth if necessary,

d) Slapping baby,

3. What can be done to prevent premature LBW newborn baby from bleeding?

a) Breastfeeding the child,

b) Not necessary to give any drugs,

c) Give Vitamin K,

d) Give Vitamin K_1,

4. Do you know what dose of Vitamin K_1 to give to a Low birth weight baby according to recommendations in the National Guidelines?

a) 0.5 mg, **b)** 1 mg, **c)** 2 mg, **d)** 5 mg,

<u>Infection management</u>

5. A premature LBW newborn child can get eye infections after delivery. Which of the following would you use to prevent this from occurring?

a) Do not apply anything,

b) Apply breast milk in the babies' eyes,

c) Clean eyes with sterile water,

d) Apply tetracycline eye ointment after cleaning eyes,

6. Taking care of the umbilical cord of a premature LBW newborn after delivery is important. Which of the following alternatives would you consider as important?

a) Always clean your hands before touching the cord,

b) Cut the cord with a clean instrument (for example, a razor blade),

c) Use any sharp instrument for cutting the cord,

d) After cutting the cord, apply traditional herbs/medicines,

7. What approach would you use to handle an umbilical cord that has any of the following signs: bad smell, oozing blood, small rashes around the umbilical area?

a) Leave to dry,

b) Clean with water and soap,

c) Clean with iodine solution,

d) Apply antibiotic powder,

<u>Special care and monitoring</u>

8. What is, in your opinion, the best way to stabilize the temperature of a premature LBW newborn baby?

a) Bathing the baby in water of appropriate temperature,

b) By putting on clothes and cover head,

c) Having the baby skin-to-skin with her/his mother,

d) Keep the baby in a room with a temperature of 28-30°C,

9. What action is important when taking care of a premature low birth weight baby immediately after birth?

a) Bath the baby often,

b) Start breastfeeding early and frequently,

c) Keep the child warm,

d) Prevent infection from developing,

10. How frequent should low birth weight babies be fed when weight is between 1250g-2500g

a) Every 2 hours

b) Every 3 hours

c) Every 4 hours

d) Every 1 hours

11. How frequent should low birth weight babies be fed when weight is less than 1250g

a) Every 2 hours

b) Every 3 hours

c) Every 4 hours

d) Every 1 hours

12. How frequent should the temperature be taken in 1500-2500 g babies in the first 24 hours

a) Every hour in the first 8hrs then every 12hrs

b) Every 6hrs then every 12hrs

c) Every 12hrs then every 24hrs

d) Every 2hrs then every 4hrs

13. How frequent should the temperature be taken in newborns below 1500 in the first 24hrs

a) Every hour in the first 8hrs then every 12hrs

b) Every 6hrs then every 12hrs

c) Every 30 min for 4hrs then hourly for 24hrs.

d) Every 2hrs then every 4hrs

14. How often should the respiratory rate be taken

a) **Every 4hrs**

b) Every 6hrs

c) Every 12hrs

d) Every 2hrs

15. How frequent should the oxygen saturations be taken?

a) Every 4hrs

b) Every 6hrs

c) Every 12hrs

d) Every 2hrs

16. How often should the heart rate be counted

a) Every 4hrs

b) Every 6hrs

c) Every 12hrs

d) Every 2hrs

17. How often should the blood glucose be taken?

a) 2hrs within delivery then 6hrly until infant getting enough feeds

b) 30mins within delivery then 12hrly until infant getting enough feeds

c) 4hrs within delivery then 24hrs until infant getting enough feeds

d) 1hr within delivery then 4hrly until infant is getting enough feeds

18. What is the acceptable daily weight gain for a premature?

a) More than 10g per day

b) More than 15g per day

c) More than 25g per day

d) More than 30g per day

19. A premature LBW is considered to have hypoglycemia when RBG is …………and should be given a bolus of…………?

a) Less than 3.3mmol/l, 5ms/kg of D10 IV stat

b) Less than 2.6mmol/l, 5ms/kg of D10 IV stat

c) Less than 3.3mmol/l, 2ms/kg of D10 IV stat

d) Less than 2.6mmol/l, 2ms/kg of D10 IV stat

20. A LBW premature admitted to your unit with fast breathing >60breaths/minute, sever chest wall in drawing, grunting and cyanosis. what is the best management for this child?

a) Put the preterm on oxygen via nasal prone

b) Put the preterm on oxygen via face mask

c) Put the preterm on CPAP

d) Put the preterm on ventilation

Supportive Information 2(S2): <u>Interview guide questions</u>

1. Can you describe the challenges you experience when providing care to premature newborns?
2. When you think about your own learning needs, what are the top 3 that you believe is most important?
3. When you consider learning needs in your workplace (unit as a whole), what do you believe is most important?
4. Can you describe what education was available for you as you entered the NICU and what ongoing education is available? For example, a scheduled orientation and ongoing professional development are scheduled regularly. Are these educational opportunities paid for as part of your position or volunteer time?
5. Can you describe or give examples of the educational opportunities offered at your institution after you started working there? Are these opportunities generally for nurses alone or in a multidisciplinary team environment?
6. There are many types of information that we need to care for ill and at-risk newborns. When you are seeking different types of information where do you look?
7. Is there a certain person (people) that you can go to within your hospital to help you find information?
8. Can you describe one or two barriers that you feel make it difficult to meet these priorities?
9. Can you describe how physicians and administration support the need for continuing professional development in your unit? How could his be improved?
10. If you could change one thing to improve your ability to provide care, what would it be?

Fig 1. social demographic characteristics

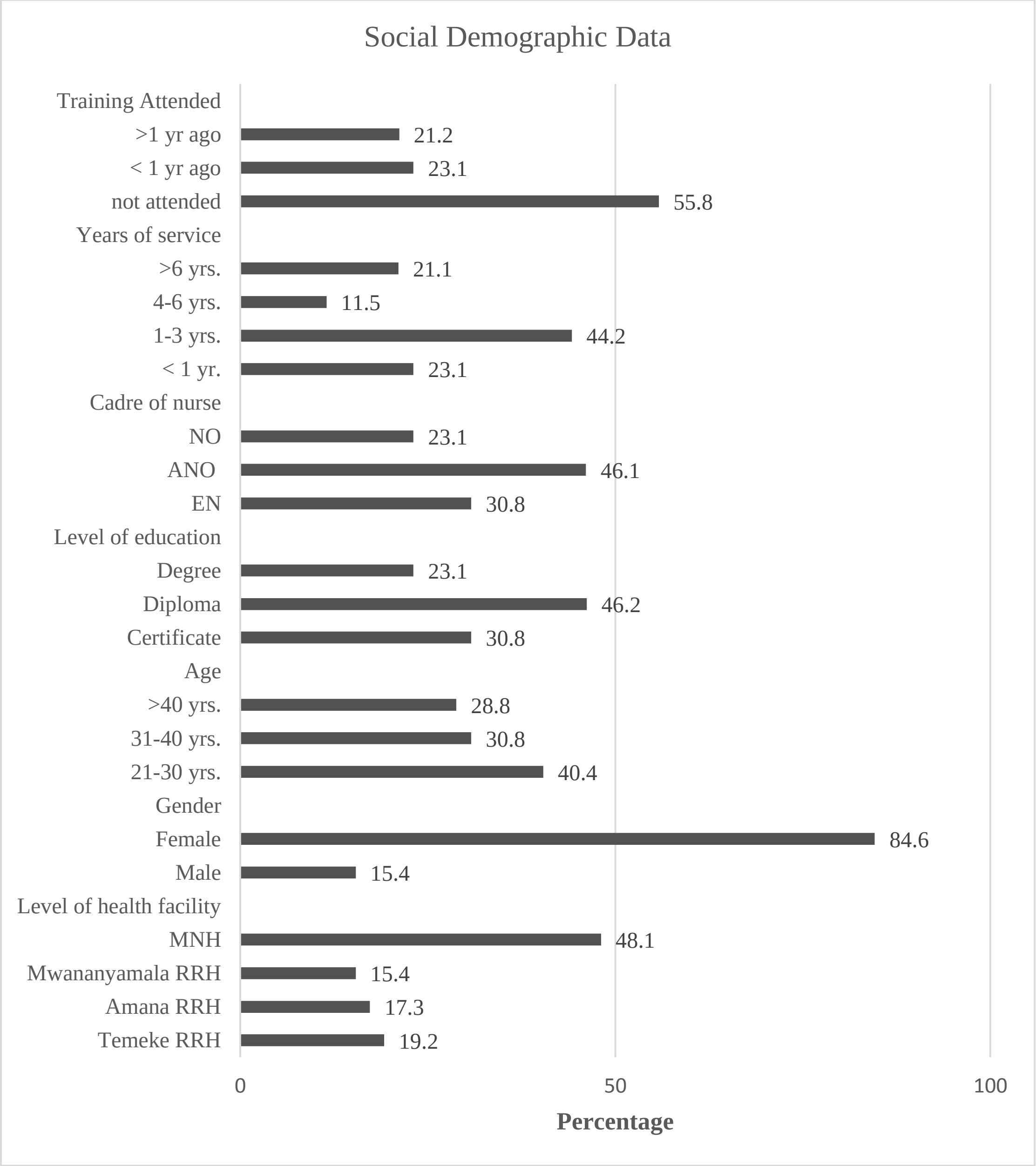

Fig 2. Nurses' knowledge on Essential New Born Care (ENC) and Infection Prevention and Management (IPM) domain by hospitals.

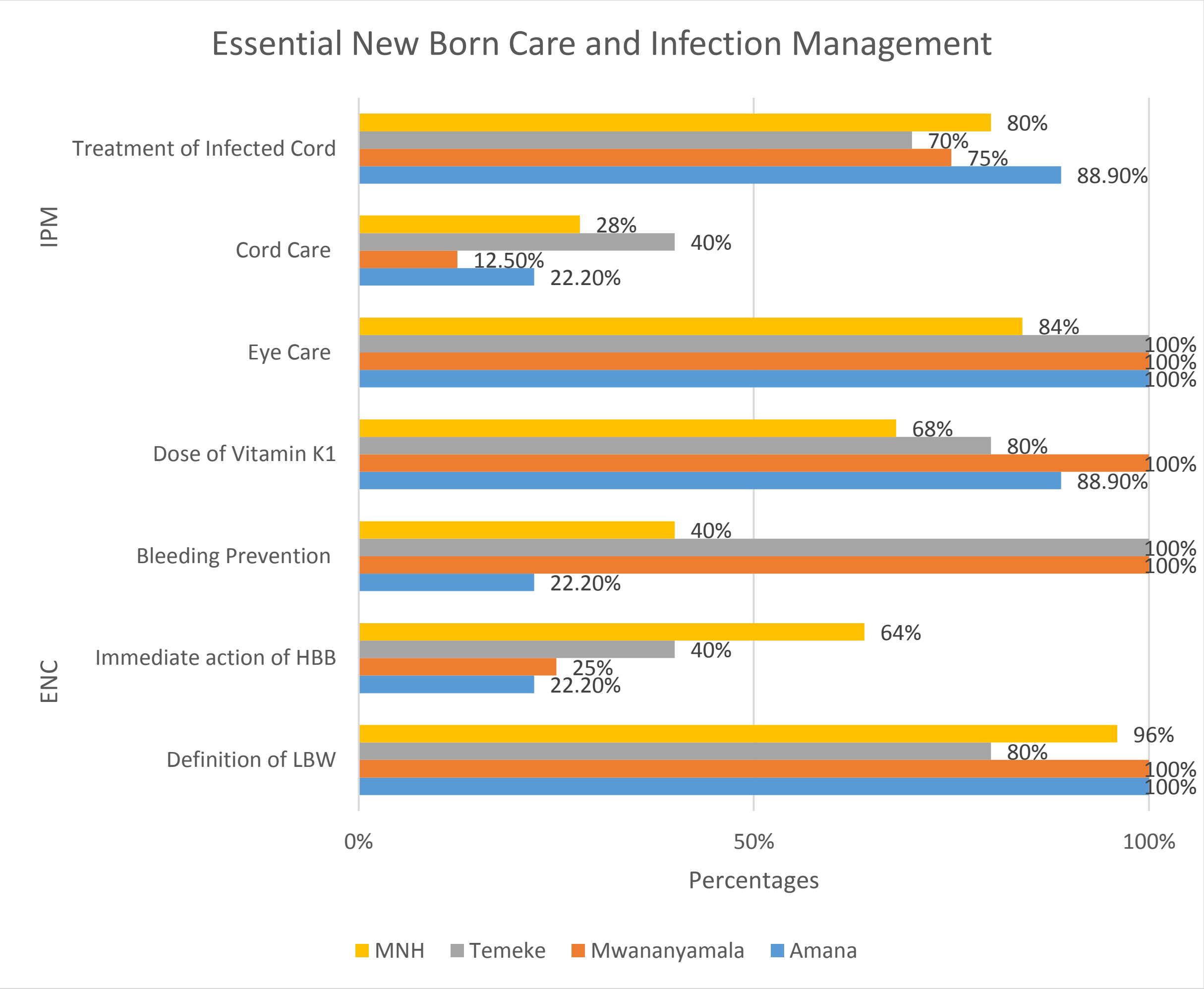

HBB-help baby breath, LBW-low birth weight

Fig 3. Nurses' knowledge on Special Care and Monitoring domains in all hospitals

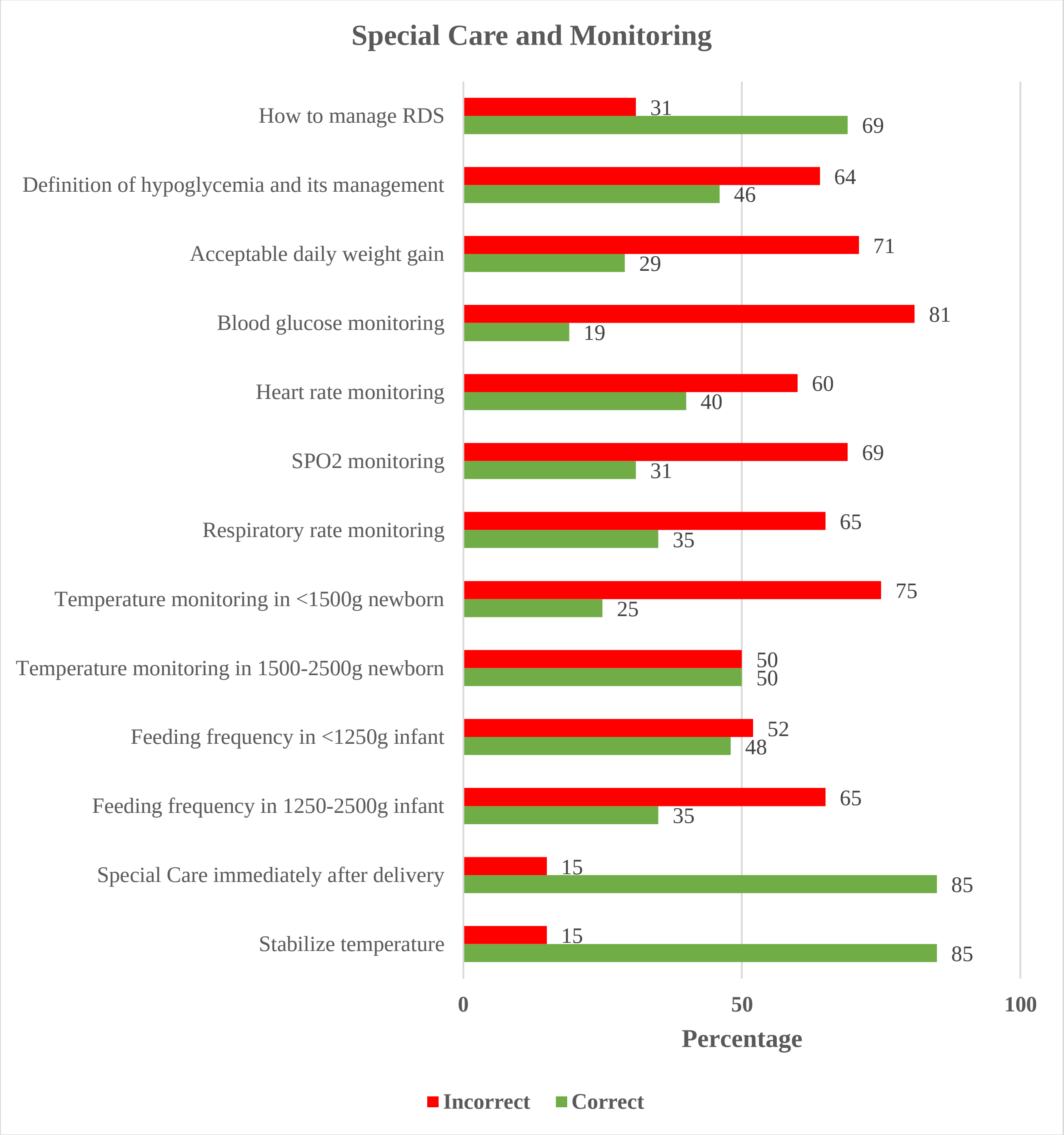

RDS- respiratory distress syndrome

Click here to access/download
Supporting Information
Supportive Information .docx
