# Supplementary material for: Nurses’ knowledge, perceived challenges, and recommended solutions regarding premature infant care: A mixed method study in the referral and tertiary hospitals in Dar es salaam, Tanzania
Source: PLoS One. 2023 Mar 29;18(3):e0281200. doi: 10.1371/journal.pone.0281200 (PMC10057798; doi:10.1371/journal.pone.0281200)
Supplement: Attachment — Submitted filename: Response to Reviewers_PLOS ONE (2).docx [file pone.0281200.s005.docx]

PONE-D-21-38982

Nurses' knowledge, perceived challenges and recommended solutions regarding premature infant care: a mixed method study in the referral and tertiary hospitals in Dar es salaam

PLOS ONE

Dear editors,

We are grateful to have received your email dated 21^st^ September 2022, containing the reviewers’ comments pertaining our manuscript titled “Nurses' knowledge, perceived challenges and recommended solutions regarding premature infant care: a mixed method study in the referral and tertiary hospitals in Dar es salaam”

We are thankful to the reviewers for taking their time to assess our manuscript. We have addressed all the concerns raised by the independent peer reviewers. Those have improved the overall quality of our manuscript.

Please find below our point-by-point response, clearly indicating how and where in the manuscript changes have been made (line numbers). To assist you in readily tracking our amendments, we included a marked up copy of the manuscript that highlights changes made to the original version. We also enclose a “clean” version of the revised manuscript as supporting information.

We very much hope that this revised version of our manuscript will now be suitable for publication in PLOS ONE. We look forward to hearing from you.

Yours Sincerely,

Dr Mwajuma Mwikali

**Academic Editor:**

The topic is quite relevant but the manuscript needs to be extensively revised as suggested by two of the reviewers who have pointed out key issues in the manuscript that needs to be addressed. Prominent amongst them is the need to provide clarity on how the sample size was determined as well as a clear presentation of the statistical analysis in the result section. The discussion should be in-depth and also harmonize the relationship between the quantitative study and qualitative study findings.

Thank you for acknowledging the relevance of the topic. The above stated corrections have been clarified as would be seen in the following upcoming comments.

**INTRODUCTION**

This section has been written as independent sentences without considering bringing these sentences with similar context as paragraphs. For example, lines 1-6 should be under one paragraph, lines 7-15 should form another paragraph.

This section is significantly deficient as it has not highlighted whether there had been previous studies on this subject in Tanzania and what justifies the need for the current study.

The whole introduction section has been rewritten by including the problem statement and rationale of the study.

**METHODOLOGY**

Page 11, line 6: and association factors

Comment: Please change to associated factors

Page 5 line 97, changed to associated factors.

Page 11, lines 13-18: This study was conducted in the three RRHs and one of the tertiary branches, MNH- Upanga. MNH-Upanga alone accounts for approximately 369 preterm deliveries per month not accounting for the referral ins (19) with bed capacity of approximately 100 in the specific premature ward. In the regional referral hospitals, preterm deliveries can be approximately 42 per month in all the hospitals (20) with a bed capacity of approximately 15 to 20 which is for both term and preterm newborns

Comment: The reason for the (19) and (20) reflected in the sentences are not clear.

Page 5 line 106-108, The reference was based on previous studies that showed the current bed state and patient capacity in the national and regional hospital.

Consider replacing the phrase “not accounting for referral ins” to “excluding the referral ins”

Page 5 line 107, The sentence has been replaced to “excluding the referral ins” as suggested.

**Sample size calculation**

The authors should clearly and explicitly describe how the sample size of 50 was derived, please also provide references for the formula and 69% proportion that was quoted. This is very important for the interpretation of the results of the study.

The sample size calculation has been revised and references included for clarity.

**Pilot study:** Where was the pilot study done, how many patients were involved in the pilot study? What were the observed issues that were corrected?

The pretest was done in the national hospital, but it was done among the nurses who work in newborn unit for the term infants, five nurses participated. Some questions were not clearly understood and stated to be vague especially the section on essential newborn care and infection prevention and management. So, the questions were reconstructed considering local context compared to the initial questionnaire from the Ugandan study.

**Sampling**: How did the authors decide on the number of nurses to be interviewed in the participating hospital? This should be highlighted

Based on our sample size calculation as explained in the methodology section. All the nurses were included except those who refused to participate.

**Qualitative study design**

Selection of nurses for qualitative study: From the regional hospital, the hospital with the most nurses with inadequate knowledge in special care and monitoring was selected.

Comment: Please explain the rationale for this step

After quantitative data collection, we performed our focus group discussion. One of the aims was to answer objective questions. We wanted to understand the challenges faced by the nurses who had inadequate response and how we can improve it. This information is essential at both, the regional and national level.

Additionally, this section has not been reported according to The Consolidated Criteria for Reporting Qualitative Studies (COREQ). This section should be systematically presented in accordance with COREQ, and the checklist must be attached as supplementary file during resubmission.

Thank you for this comment. The section was rewritten based on the COREQ checklist and it was included in the supplementary file as per standard required.

**RESULTS**

This section, particularly the qualitative component, has not been well presented. There is need for a univariate analysis before multiple regression analysis in the quantitative arm of the presentation of findings while the presentation of findings following the themes that emanated from the FDGs are quite scanty for comprehension by readers.

The analysis tables were constructed to include the step-by-step process from univariate to multivariate analysis. The results for qualitative study was rewritten to be more comprehensive.

**DISCUSSION**

Overall, this section has been poorly written without considering the essence of the mixed methods study design. There is no effort at triangulation of results and there has not been any in-depth discussion of the study’s findings. In the quantitative arm, the studies used to compare findings were limited. For example, knowledge on essential newborn care (ENC): There is a fault in comparing two studies with different study populations. While the current study was conducted amongst nurses involved in the management of premature babies, the study in Uganda was carried out amongst outpatient nurses. Therefore, it is not surprising to have a higher knowledge in the former group. Please rewrite this section.

The section was rewritten with the efforts of triangulation of the results in the two studies. There were few studies done to compare our results with, but we have managed to make changes the best we could.

**Study limitations:** Please write as prose and delete item 3.

Changed to prose and item 3 deleted

**Reviewer #1:**

It is preferable to know more about the setting that the study was conducted in and definitions of prematurity

We have been able to describe the setting of the hospitals the study was conducted at the opening statement of the methodology section. The definition of prematurity has been included at the beginning of the introduction section.

The questionnaire is very limited. For example, there is much more to Infection control than cord care.

The questionnaire was adapted from previous study done in Uganda then modified some questions based on preterm infant nursing care in our national neonatal guideline. We didn’t consider other ways of infection prevention and management elements.

I do not feel that it adds much to the discussion to compare the results from this study to similar ones conducted in other countries.

Discussion section has been improved by mainly focusing on triangulation of results from the 2 methods used. Thank you for the input. Has helped in improving the write up.

**Reviewer #2:** 

This manuscript is addressing a very topical and an important subject matter. However, the following should be clarified.

Thank you for appreciating the importance of this study.

The use of symbols is not ideal in writing.

Thank you for that correction. It has been rectified

In the methodology, the sample size calculation is not clearly stated. The assumptions made in the calculation of sample size were not clarified. The research assistants were not clearly defined. Are they nurses in the hospitals used for the study or not? The statistical analysis was not explained detailed. How the adjusted odds ratio was generated was not clear. There ought to be a bivariate analysis of the different domains before a multivariate analysis. This was not the case in this manuscript.

We have managed to clearly elaborate how we came up with our sample size as requested.

Explanation on the study population has been stated together with how the research assistants were attained.

New tables have been formed that are clear on how the analysis was done step by step.

Table 1 is very clumsy due to the observation stated in 2 above.

The tables have been edited to less clumsy ones

In the discussion, there was no triangulation of the quantitative and qualitative study. It thus appears they were different studies. Ideally there should be a link between both in the discussion. The discussion should be re-written in a more robust manner.

Discussion session has been rewritten with inclusion on triangulation of the two methods. Thank you for that input that has enable improvement in the manuscript

**Reviewer #3:**

This article addresses an important subject of improving the survival of premature neonates, through improving the care they receive while in hospital. The authors have presented an article that has potential but will require several modifications.

Thank you for acknowledging the importance of this article. Modification on the methodology write up, results and discussion have been made.
